# Diagnostic and Therapeutic Challenges of Homozygous and Severe Heterozygous Familial Hypercholesterolemia from Clinical Aspect—A Single-Center Study

**DOI:** 10.3390/jcm14228058

**Published:** 2025-11-13

**Authors:** Bíborka Nádró, Judit Kaluha, Hajnalka Lőrincz, Éva Varga, István Balogh, Mariann Harangi

**Affiliations:** 1 Division of Metabolic Diseases, Department of Internal Medicine, Faculty of Medicine, University of Debrecen, 4032 Debrecen, Hungary; 2 Department of Internal Medicine and Hematology, Semmelweis University, 1083 Budapest, Hungary; 3 Department of Medical Genetics, Faculty of Medicine, University of Debrecen, 4032 Debrecen, Hungary; 4 ELKH-UD Vascular Pathophysiology Research Group 11003, University of Debrecen, 4032 Debrecen, Hungary; 5 Institute of Health Studies, Faculty of Health Sciences, University of Debrecen, 4032 Debrecen, Hungary

**Keywords:** familial hypercholesterolemia, homozygous, heterozygous, low-density lipoprotein cholesterol, genetic testing, lipid-lowering therapies, therapy resistance, personalized medicine

## Abstract

**Background/Objectives:** The clinical presentation of homozygous familial hypercholesterolemia (HoFH) and severe heterozygous familial hypercholesterolemia (sHeFH) often demonstrates substantial overlap, as low-density lipoprotein cholesterol (LDL-C) levels may fall within similar ranges in both conditions. **Methods:** In this single-center 10-year retrospective study at the University of Debrecen, Hungary, we present the clinical characteristics of patients with 6 HoFH and 6 sHeFH diagnosed by genetic testing, discuss the diagnostic limitations encountered in clinical practice, and outline the key components of therapeutic management. **Results:** The mean age at diagnosis was lower in the HoFH group (31.83 ± 19.5 vs. 41.83 ± 15.9 years). The differences in total cholesterol (13.48 ± 7.4 vs. 11.02 ± 3.5 mmol/L) and LDL-C levels (10.89 ± 6.6 vs. 8.58 ± 3.26 mmol/L) between the groups were not statistically significant. Interestingly, vascular complications were more frequent in sHeFH group as well (4 vs. 1 patients). In neither the HoFH nor the sHeFH group were we able to achieve the target LDL-C levels, due in part to the specific features of the reimbursement system, patient and parental preferences, the extremely high baseline LDL-C levels, and certain genetic characteristics. **Conclusions:** Our findings highlight the importance of genetic testing-based personalized therapy in these specific patient subpopulations. We emphasize that serum LDL-C alone is insufficient to distinguish between HoFH and sHeFH patients, and that therapeutic challenges should be anticipated in both groups arising partly from limited patient adherence as well as from financial constraints.

## 1. Introduction

The heterozygous form of familial hypercholesterolemia (HeFH) is one of the most common monogenic inherited metabolic disorders, with an estimated worldwide prevalence of 1:300 [1]. In Hungary, a previous study reported a prevalence of approximately 1:340 [2]. The more severe, homozygous form (HoFH) is rare, the prevalence had been estimated as 1 in 1,000,000 before but more recent genetic analysis surveys predict 1 in 170,000 to 300,000 [3]. Familial hypercholesterolemia is characterized by the presence of one (HeFH) or two (HoFH) pathogenic variants in the low-density lipoprotein receptor (*LDLR*), apolipoprotein B (*APOB*), proprotein convertase subtilisin/kexin type 9 (*PCSK9*) or LDL-receptor adaptor protein 1 (*LDLRAP1*) genes, which cause very high levels of low-density lipoprotein (LDL) cholesterol (LDL-C) and premature atherosclerotic cardiovascular disease. In HeFH, total cholesterol and LDL-C vary widely, and in certain cases, significantly elevated values are found, which can lead to the development of early vascular complications [4]. Indeed, the risk of developing cardiovascular disease in HeFH patients is estimated to be 3 to 13 times higher compared to the general population [5,6]. Although less frequently than in HoFH, external manifestations such as xanthomas, xanthelasmata and corneal arcus may also occur. In these severe HeFH cases (sHeFH), markedly elevated serum LDL-C, accelerated atherosclerosis and early vascular complications are characteristic. By the age of 40, nearly 90% of patients with HoFH have already experienced a cardiovascular event [7]. Clinical presentation and variations in serum lipids can be significantly influenced by other genetic factors (such as polymorphisms and co-existing mutations), environmental factors, and comorbidities [8,9,10]. These elements may explain the phenotypic similarities between the two patient groups. However, the clinical assessment and treatment strategies for the two conditions substantially differ, making early diagnosis critically important. For patients with HeFH, treatment typically begins with statins, bempedoic acid and ezetimibe, followed—if the response is inadequate—by proprotein convertase subtilisin/kexin type 9 (PCSK9) inhibitors. Selective LDL apheresis is required only in selected cases [11]. In patients with HeFH, the recommended LDL-C targets are <3.5 mmol/L for children, <2.5 mmol/L for adults, and <1.8 mmol/L for adults with established coronary heart disease or type 2 diabetes [12,13]. Barriers to achieving target lipid levels may arise from therapeutic inertia among clinicians, poor treatment adherence among patients, and limited willingness of healthcare authorities to fund more expensive therapeutic options [14]. In contrast, for patients with HoFH, if the combination of statin and ezetimibe plus a PCSK9 inhibitor is not sufficiently effective, treatment should include agents with LDL receptor-independent mechanisms of action, such as the microsomal transfer protein (MTP) inhibitor lomitapide or the angiopoietin-like protein 3 (ANGPTL3) inhibitor evinacumab, often in combination with selective LDL apheresis [15]. In adults with HoFH, the target LDL-C is <1.8 mmol/L or <1.4 mmol/L in the presence of an additional cardiovascular risk factor, such as elevated lipoprotein(a) (Lp(a)), type 2 diabetes mellitus or established atherosclerotic cardiovascular disease. For children and adolescents an LDL-C target of <3 mmol/L is recommended when therapy is initiated before the age of 18 and imaging studies show no evidence of atherosclerotic cardiovascular disease [15]. In HoFH achieving lipid targets lipid remains challenging even with the combined use of these effective novel therapeutic options. Moreover, in many parts of the world, the availability or reimbursement of these treatments is restricted due to high cost [15]. The basis for diagnosis is genetic testing [4], although the results do not always correlate with the severity of lipid abnormalities or the presence of symptoms, which significantly complicates the work of healthcare professionals responsible for patient care.

We aimed to present two patient groups—one with HoFH and one with sHeFH—highlighting the diagnostic and therapeutic challenges and emphasizing the importance of developing personalized treatment strategies. Our aim was to compare the characteristics of genetically confirmed HoFH and selected sHeFH patients treated at the Lipidology Center of the University of Debrecen between 2015 and 2025 based on their lipid parameters, clinical features and cardiovascular complications. Additionally, we aimed to evaluate their treatment regimens, the achievement of target LDL-C and the limitations associated with attaining these goals. We hypothesized that there would be no significant differences between the two study groups in serum LDL-C, the prevalence of cardiovascular complications, or external manifestations. Moreover, we hypothesized that the proportion of patients achieving LDL-C targets would be higher in the sHeFH group.

## 2. Materials and Methods

### 2.1. Patients

Among patients with familial hypercholesterolemia treated at our University’s Lipidology Center between 2015 and 2025, genetic testing is performed in certain cases, such as those with severe clinical presentations, childhood forms or therapeutic decision-making challenges. Based on our clinical database, we enrolled 6 patients with HoFH (all HoFH patients diagnosed in our center) and 6 patients with sHeFH (selected to the other patient group based on clinical and laboratory parameters). The phenotype is severe when LDL-C levels exceed three to four times the normal level, and external or cardiovascular manifestations of FH are present [16]. For HoFH, the inclusion criterion was the presence of two pathogenic mutations confirmed by genetic testing in the *LDLR, ApoB100, PCSK9*, or *LDLRAP1* genes. All six genetically confirmed HoFH patients were enrolled. In sHeFH, the inclusion criterion was the presence of one pathogenic mutation in any of the aforementioned genes, confirmed by genetic testing. Additionally, serum LDL-C was required to exceed the normal range by three to four times, or the presence of either external or cardiovascular manifestations had to be observed [16]. Among the 13 sHeFH patients, six with the most severe laboratory and clinical profiles were enrolled. In both groups, exclusion criteria were the absence of genetic testing, incomplete laboratory or clinical data and lack of patient consent. Dutch Lipid Clinic Network (DLCN) scores were calculated and resulted in “definite FH” in all cases [17]. Patients were recruited from the Lipid Outpatient Clinic of the Department of Internal Medicine, University of Debrecen. All patients were confirmed by genetic testing. The patients were referred to our Lipid Outpatient Clinic by GPs and other specialists, such as cardiologists and neurologists, to verify the diagnosis of familial hypercholesterolemia and initiate optimal therapy. These were scheduled medical appointments from 08:00 to 10:00 AM and we asked the patients to arrive after 12 h of fasting. Most of the patients were newly diagnosed without ongoing lipid-lowering medical treatment. The medical history of each patient was reviewed to identify previously diagnosed vascular complications including acute myocardial infarction, ischemic stroke, carotid artery disease or peripheral arterial disease (PAD). Furthermore, in all patients, a thorough medical history was obtained, and physical examination, resting electrocardiography, transthoracic echocardiography, and carotid artery Doppler ultrasonography were performed. In selected cases, additional investigations such as exercise electrocardiography, coronary angiography, or lower limb arterial Doppler ultrasonography were also carried out as indicated. The study design is illustrated in Supplementary Figure S1.

All participants, or in the case of children, their legal guardians provided written informed consent. The study protocol was approved by the local and regional ethical committees (DE RKEB/IKEB 4775-2017, date obtained: 3 April 2020 and ETT TUKEB 34952-1/2017/EKU, date obtained: 30 June 2017) and the study was carried out in accordance with the Declaration of Helsinki.

### 2.2. Blood Sampling

Venous blood samples were drawn after overnight fasting, and sera were prepared immediately. Routine laboratory investigations (triglyceride, total cholesterol, LDL-C, high-density lipoprotein cholesterol (HDL-C), apolipoprotein B100 (ApoB100), apolipoprotein A1 (ApoA1) and Lp(a)) were carried out on the fresh sera using a Cobas c600 autoanalyzer (Roche Diagnostics GmbH, Mannheim, Germany) in the Department of Laboratory Medicine, Faculty of Medicine, University of Debrecen, Hungary. The reagents were purchased from the same vendor and the tests were performed according to the recommendations of the manufacturer.

### 2.3. Sequencing of Genes Related to Familial Hypercholesterolemia (LDLR, APOB, PCSK9, LDLRAP1)

Genes related to familial hypercholesterolemia were analyzed using the Devyser FH v2 kit (Devyser AB, Hägersten, Sweden). This amplicon-based library preparation kit can examine the coding region and exon/intron boundaries (minimum +/−10 base pairs) of the following genes: *APOB*: NM_000384.3, *LDLR*: NM_000527.5, PCSK9: NM_174936.4, *LDLRAP1*: NM_015627.3. Sequencing was performed on Illumina MiSeq with 300 cycles according to the manufacturer’s protocol. Raw data analysis was carried out with the NextGENe v2.4.2.3 (SoftGenetics, State College, PA, USA) software, with a minimum coverage requirement of 40×. Copy number variation detection was performed based on coverage data. The detected pathogenic variants were confirmed by Sanger sequencing. Primers were designed using Primer3 v2.5.0 software (https://primer3.ut.ee/) (accessed on 15 October 2021). Primers are listed in the paper of Madar et al. [18].

### 2.4. Multiplex Ligation-Dependent Probe Amplification (MLPA)

Confirmation of a copy number variation detected by next-generation sequencing was performed in three cases by MLPA method using SALSA MLPA Probemix P062 Mix 1 (MRC Holland, Amsterdam, The Netherlands) according to the manufacturer’s protocol [18].

### 2.5. Variant Filtering and Interpretation

All detected variants with an MAF > 0.01 (minor allele frequency) in the gnomAD population database were filtered. The remaining variants were classified according to the ACMG standards and guidelines [19]. A web-based interpretation tool, Franklin (Genoox, Tel Aviv, Israel), was used to assist the classification. HGMD Professional and ClinVar databases were also used in variant interpretation.

### 2.6. Statistical Analysis

Statistical analyses were performed using the Statistica 13.5.0.17 software (TIBCO Software Inc., Palo Alto, CA, USA). Graphs were made by GraphPad Prism 8.01 (GraphPad Prism Software Inc., San Diego, CA, USA). Normality of variables was checked using the Kormogorov–Smirnov test. Continuous variables were expressed as median (interquartile ranges) or mean (standard deviation). Comparisons between groups were performed by Student’s unpaired *t*-test in case of normally distributed variables and by the Mann–Whitney U-test in case of variables with non-normal distribution.

## 3. Results

**Figure 1:**
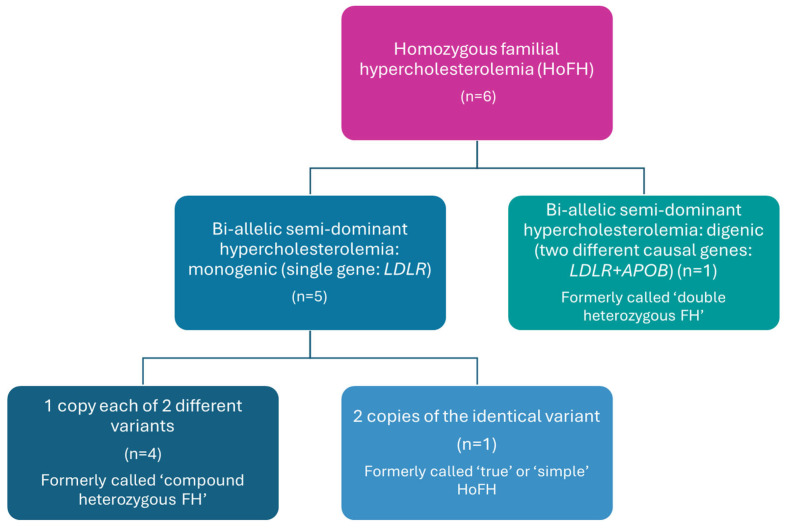
Types of genetic abnormalities in patients with homozygous familial hypercholesterolemia. APOB: apolipoprotein B; HoFH: homozygous familial hypercholesterolemia; FH, familial hypercholesterolemia; LDLR: low-density lipoprotein receptor.

Characteristics and laboratory parameters of HoFH patients at diagnosis are summarized in Table 1. At the time of diagnosis, all patients exhibited extremely high total cholesterol and LDL-C; however, these values varied widely regardless of age. The highest serum LDL-C was observed in the child with true homozygosity with two alleles of a known pathogenic *LDLR* mutation. Although elevated serum triglycerides are not characteristic of HoFH, moderately increased values were observed in the two youngest patients. There were also notable differences in HDL-C. Interestingly, none of the patients showed external manifestations—no xanthomas, xanthelasmata or arcus corneae were detected. Compared to data in the literature, the incidence of ischemic heart disease was surprisingly low, with only one confirmed case, and no other vascular complications were identified despite active screening. Genetic testing revealed the presence of two different pathogenic mutations in most cases, typically in the *LDLR* gene (compound heterozygous); in one case, one pathogenic mutation was found in *LDLR* and another in *APOB* (double heterozygous). No *PCSK9* gene mutations were detected in any of our cases. DLCN scores resulted in “definite FH” in all cases. A positive result of the DNA analysis for a functional mutation in either one of the *LDLR*, *ApoB100* or *PCSK9* genes equals +8 points (Table 1, Figure 1).

Following the establishment of the diagnosis, all patients—or in the case of children, their legal guardians—were provided with detailed information regarding the etiology and characteristics of the disease, its hereditary nature, potential complications, and available treatment options. This counseling was conducted with the involvement of a clinical geneticist and a lipid specialist. In all cases, initiation of statin therapy was recommended in accordance with the Hungarian clinical guidelines. Even at this early stage, we observed resistance to pharmacological treatment in several cases, particularly among younger patients (Patients 2, 3, and 4) and their parents. This reluctance was primarily attributed to the absence of symptoms and concerns about potential side effects. The parents of Patient 2 subsequently discontinued follow-up and did not return for further care. In the cases of Patients 3 and 4, a fixed-dose combination therapy of atorvastatin 20 mg and ezetimibe 10 mg daily resulted in a significant reduction in LDL-C; however, both patients declined the addition of PCSK9 inhibitor therapy. Patient 1 is currently receiving combination therapy with rosuvastatin 40 mg and ezetimibe 10 mg daily, along with alirocumab 150 mg biweekly, with good therapeutic efficacy. In the case of Patient 5, complete statin intolerance was confirmed. The patient is being treated with ezetimibe 10 mg daily and alirocumab 150 mg biweekly, which has shown moderate efficacy. Therefore, we plan to complement the treatment with evinacumab. However, similarly to several other countries, the approval process for this therapy involves a complicated administrative procedure. Patient 6 was diagnosed a few months ago. Given the limited efficacy of the current regimen (atorvastatin 20 mg and ezetimibe 10 mg daily) the patient is scheduled to undergo selective LDL apheresis, contingent upon establishing vascular access. In parallel, enrollment in a lomitapide treatment program is currently underway. To date, none of the above-mentioned patients could achieve LDL-C target.

**Figure 2:**
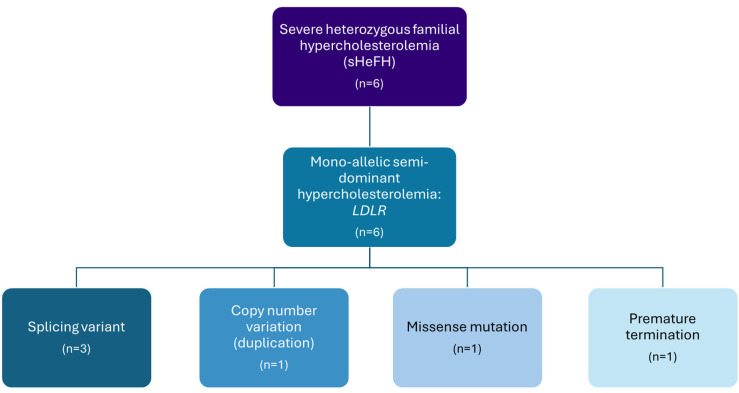
Types of genetic abnormalities in patients with severe heterozygous familial hypercholesterolemia. sHeFH: severe heterozygous familial hypercholesterolemia; LDLR: low-density lipoprotein receptor.

Table 2 presents the most relevant clinical and laboratory data of the sHeFH patients. Compared to the majority of patients with HeFH, those included in this study generally exhibited higher total cholesterol and LDL-C. In contrast to other patients of this group, total and LDL-C concentrations of Patients 8 and 11 were only moderately elevated; however, early-onset and severe cardiovascular complications were observed. Serum HDL-C fell within the normal range in all patients, while four individuals had triglyceride concentrations exceeding the recommended target values. Tendinous xanthoma was documented in one case, and xanthelasma of the eyelids was noted in another. In two patients, in addition to severe coronary artery involvement, extensive atherosclerosis was also confirmed in other vascular regions. In all cases, a known pathogenic mutation was identified in the *LDLR* gene. These included three splicing variants, one duplication affecting four exons, one nonsense mutation resulting in a premature stop codon, and one missense mutation. DLCN scores resulted in “definite FH” in all cases. A positive result of the DNA analysis for a functional mutation in either one of the *LDLR*, *ApoB100* or *PCSK9* genes equals +8 points (Table 2, Figure 2).

Similarly to the approach taken in HoFH cases, following diagnosis, patients—and, in the case of pediatric patients, their guardians—received detailed counseling provided by a clinical geneticist and an experienced lipidologist. This counseling covered the disease course, potential complications, and available therapeutic options. Patient 7 was treated with ezetimibe and subsequently with a PCSK9 monoclonal antibody (alirocumab) due to statin-induced myopathy and complete statin intolerance. However, neither therapy resulted in a clinically meaningful reduction in the patient’s extremely elevated LDL-C. A similar lack of therapeutic efficacy was observed after the administration of inclisiran. The suboptimal response may have been due to an exon duplication involving the PCSK9 binding domain. As a result, the patient underwent selective LDL apheresis; however, the LDL-C target was still not achieved. In Patient 8, treatment consisted of daily ezetimibe (10 mg) and rosuvastatin (40 mg), along with biannual subcutaneous inclisiran (284 mg). This regimen led to a marked reduction in LDL-C levels; however, target values were still not reached. In the case of Patient 9, a child presenting with extremely elevated LDL-C, a premature stop codon in the *LDLR* gene was identified as the underlying genetic cause. Lipid-lowering therapy was initiated under the supervision of a pediatric specialist. However, even at low doses, statin therapy induced myopathic symptoms, leading to the initiation of ezetimibe monotherapy. The patient’s parents are currently considering treatment with a PCSK9 monoclonal antibody, although they remain hesitant about this therapeutic option. Patient 10 is also statin-intolerant due to statin-induced myopathy. As target LDL-C is unlikely to be achieved with ezetimibe monotherapy alone, the initiation of a PCSK9 inhibitor is clinically warranted. However, current reimbursement policies do not support the use of these agents for primary prevention. Consequently, an individual request for reimbursement has been submitted to the national health insurance authority; the application is currently under review. In the case of Patient 11, a young adult male with partial statin intolerance due to statin-induced myopathy, a tolerable regimen was established consisting of 10 mg ezetimibe daily and 10 mg rosuvastatin administered on alternate days. Inclisiran therapy (284 mg subcutaneous injection) was initiated but proved ineffective, despite the lack of a mechanistic explanation from the identified point mutation in exon 12. Should PCSK9 monoclonal antibody therapy also fail to yield adequate LDL-C reduction, initiation of LDL apheresis may need to be considered. However, the patient, who leads an active and professionally engaged lifestyle, is currently unwilling to pursue this option. Patient 12 presented with tendon xanthomas and a history of Achilles tendon rupture, in addition to confirmed generalized atherosclerotic vascular disease and markedly elevated baseline total cholesterol and LDL-C. Due to an inadequate response to 10 mg ezetimibe and 40 mg rosuvastatin daily, monthly LDL apheresis was initiated over a decade ago. Following the appearance of PCSK9 inhibitors, apheresis was successfully discontinued. The patient is currently on maximum-dose fixed combination therapy with ezetimibe and rosuvastatin, supplemented by subcutaneous evolocumab 140 mg every two weeks, under which LDL-C target levels have been neared, but not achieved.

Comparison of the HoFH and sHeFH patient groups is presented in Table 3. The mean age at diagnosis was somewhat lower in the HoFH group. However, the differences in total cholesterol, LDL-C and ApoB100 between the groups were not statistically significant. Median Lp(a) was slightly higher in the HoFH group compared to the HeFH patients, but the difference did not reach statistical significance. There were no statistically significant differences between the two groups in any other lipid parameters. Interestingly, tendon xanthomas were observed exclusively in the sHeFH group, and vascular complications were also more frequent in this group, but the differences were not statistically significant.

**Figure 3:**
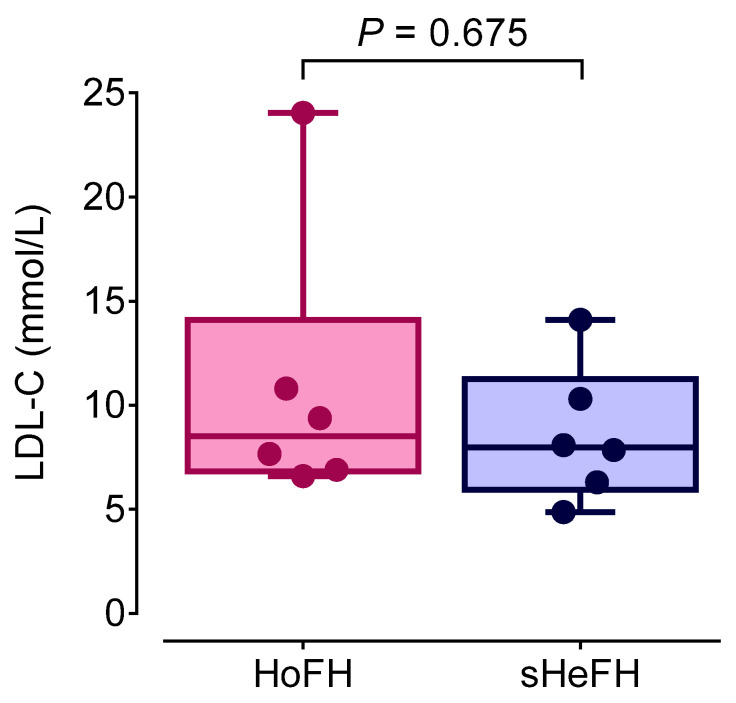
Serum LDL-C levels in HoFH and sHeFH patients. HoFH: homozygous familial hypercholesterolemia, sHeFH: severe heterozygous familial hypercholesterolemia; LDL-C: low-density lipoprotein cholesterol.

Although the highest LDL-C value was detected in the HoFH group, there is a significant overlap between the serum LDL-C levels of HoFH and sHeFH patients. There was no significant difference in serum LDL-C levels between HoFH and sHeFH patients (Figure 3).

## 4. Discussion

This is the first head-to-head comparison of HoFH and sHeFH patients to provide real-world data on these unique patient populations. The HoFH and sHeFH patients presented in this retrospective single-center study illustrate the substantial overlap between clinical features and laboratory parameters of these two cohorts. Furthermore, the results underscore the therapeutic challenges encountered in clinical practice, which are partly attributable to reimbursement and healthcare system limitations. Additionally, gaps in patient knowledge regarding the nature of the disease and available therapeutic options were evident. These factors significantly impact patient adherence and engagement, thereby influencing the overall effectiveness and long-term success of treatment strategies.

In line with previously published international data, the condition is often diagnosed at a late stage in Hungarian patients as well, resulting in a delayed initiation of effective treatment. According to data from the European Atherosclerosis Society Familial Hypercholesterolaemia Studies Collaboration (FHSC) global registry, among 61,612 registered individuals with HeFH, the median age at diagnosis was 44.4 years, with 40.2% of participants being younger than 40 years at the time of diagnosis [20]. In our own cohort of sHeFH patients, the mean age at diagnosis was in line with the literature data. Data from 389 women and 362 men with HoFH across 38 countries showed a similar age at diagnosis between sexes (13 years for women vs. 11 years for men) [21]. According to the Spanish registry, the mean age at diagnosis was 14 years [22]. In contrast, among our HoFH patients, the diagnosis was established at a significantly later age, approximately 8 years earlier than in our sHeFH group, yet still markedly longer than the international data. This delay suggests suboptimal early detection of HoFH in Hungary. Potential contributing factors include limited awareness of the disease among both the general population and healthcare professionals, restricted access to advanced laboratory diagnostics, and the absence of systematic screening programs. Indeed, our country joined ScreenPro FH in the 2000s. This project aimed to improve the identification, diagnostics, and treatment of FH patients in the regions of Central, Eastern, and Southern Europe. Although there are some improvements, the screening activity should be enhanced in this region [23].

Based on international data, the mean LDL-C level in HoFH patients is approximately 15.2 mmol/L [21]. In our cohort, the average LDL-C level was slightly lower. This difference is most likely attributable to the underlying genetic variation in our patient population. Since we selected severe HeFH cases, the mean LDL-C level of our sHeFH patients is significantly higher compared to a general HeFH population, highlighting the presence of a special subpopulation within HeFH patients. The overlap in LDL-C between the HoFH and HeFH patient groups highlights that differentiation between the two conditions cannot be reliably achieved based on LDL-C concentrations alone, due to the genetic heterogeneity of both patient groups. Without genetic testing, an accurate diagnosis cannot be established. The absence of xanthomas in the majority of patients also represents a diagnostic challenge and further emphasizes the importance of genetic testing. While the DLCN scoring system provides valuable support in identifying probable and definite cases of FH, HoFH may still be present among patients categorized as having “Probable FH”, and such cases can only be confirmed by genetic analysis. The applicability of the DLCN score is particularly limited in young patients [24,25]. The elevated Lp(a) observed in both groups is consistent with previous literature [26,27]. Previous studies have also shown that serum Lp(a) is higher in HoFH patients compared to those with HeFH [28]. This trend was also observed in our cohort; however, the difference did not reach statistical significance, likely due to small sample size.

Performing genetic testing in this subpopulation is essential for differential diagnostic purposes. Moreover, it may help to better understand the severity of the clinical phenotype. A previously published Italian study enrolled 125 homozygotes with autosomal dominant hypercholesterolemia. Approximately 98% of them carried (likely)/pathogenic variants of the *LDLR* gene [29]. In line with the international data, *LDLR* mutations were identified among our HoFH patients, and only one double heterozygous (*LDLR* and *APOB*) patient was identified. As illustrated by the presented cases, sHeFH patients in this group often exhibit splicing variants, premature stop codons, or even exon duplications or deletions, rather than single amino acid substitutions caused by missense mutations. In contrast, previous studies have shown that patients with HeFH predominantly carry missense mutations resulting from single nucleotide substitutions [18]. Our findings underline the pivotal role of genetic testing in diagnostics, as LDL-C concentrations alone are insufficient to distinguish between HoFH and severe HeFH.

Tendon xanthomas are pathognomonic for both HeFH and HoFH, but not apparent during childhood and gradually appear around puberty. Moreover, xanthomas can be repressed or made to regress with continuous aggressive lipid-lowering treatment [3]. A previous study found that HeFH patients with pathogenic FH mutations exhibited xanthomas at 6.3% and corneal arcus at 4.2% [30]. In a Spanish study including patients or their relatives with genetically proven heterozygous FH, xanthomas were detected in 15.2% [31]. In our cohorts, only one sHeFH patient had tendon xanthoma. In must be noted that numerous factors such as ethnicity, definition of xanthomas (location, thickness, method of measurement), and pre-treatment with lipid-lowering medication may have contributed to the observed differences [30].

According to data from the FHSC registry, vascular complications are not uncommon even in HeFH. The reported prevalence rates were 17.4% for coronary artery disease (CAD), including 11.3% for premature CAD, 5.2% for PAD, and 2.1% for stroke [32]. Similarly, in the sHeFH population we studied, the prevalence of vascular complications was also high. In line with the FHSC data, CAD was the most frequent vascular event observed in our cohort. However, the prevalence of vascular complications in the HoFH group was surprisingly low, which can be explained by the young age of this population.

Based on the data of genetically confirmed patients with HoFH of the CASCADE FH Registry, even those who are diagnosed often fail to achieve optimal LDL-C levels despite multiple therapies. Most of the patients either were on no lipid-lowering treatment (40%) or were grossly undertreated [33]. Delayed treatment of pediatric HeFH seems to be a persistent problem worldwide as well. In a Norwegian cohort of 302 HeFH children lipid-lowering therapy was initiated at a mean age of 12.5 years, 58% of the cohort were treated, and only 43% of patients achieved the LDL-C goal [34]. According to the data of a retrospective and prospective multicenter cohort study 674 pediatric patients with HeFH followed up in specialist lipid clinics in France received late and suboptimal treatment and half of them do not reach the therapeutic LDL-C goal [35]. Based on the recently published data of the FHSC registry, among the enrolled 10,428 HeFH children or adolescents, 7557 (72.4%) were not taking lipid-lowering medication and had a median LDL-C of 5.00 mmol/L [32].

Our own experience is consistent with the above-mentioned international data: in neither the HoFH nor the sHeFH group were we able to achieve the target LDL-C levels recommended by clinical guidelines [13,15], due in part to the reimbursement system, patient and parental preferences, extremely high baseline LDL-C concentrations, and certain genetic characteristics. Although the necessity of early and aggressive LDL-C reduction is evident in both HoFH and sHeFH, this cannot be achieved with conventional therapeutic agents. Even the new, highly effective drugs do not provide a solution in the majority of cases. Lomitapide is a powerful drug available for the treatment of HoFH. Its long-term efficacy has been proved by an open-labeled Japanese trial [36]. Evinacumab is a new treatment option for both adult and pediatric patients with HoFH and inadequately controlled LDL-C despite optimized lipid-lowering therapy, lowering LDL-C levels by nearly half in these extremely high-risk and difficult-to-treat individuals [37]. However, high therapeutic cost and limited availability currently restrict the applicability of these effective and safe agents in most countries worldwide.

A key strength of our study is that it draws attention to the numerous similarities between patients with HoFH and those with sHeFH, while also highlighting the diagnostic and therapeutic challenges associated with these conditions. Our findings underscore that adherence to clinical guidelines in everyday medical practice faces multiple obstacles, which clearly hampers the effective treatment of patients. In this context, the presentation of real-world data related to these practical challenges can be particularly valuable for practicing physicians. Among the limitations of our study, we must mention the single-center retrospective design of the study, the heterogeneous follow-up, and the relatively small number of enrolled patients, which reflects the low prevalence of HoFH, as the number of sHeFH patients was determined in consideration of the HoFH patients. Indeed, the sHeFH group does not represent the broader HeFH population; rather, it was intentionally composed of the most severe cases, highlighting the greatest therapeutic challenges. Therefore, the comparison between these two groups does not reflect the general characteristics of HoFH and HeFH patients. Instead, it emphasizes the similarities between patient groups with questionable differential diagnostic classification. Another limitation is the genetic and clinical heterogeneity of the two studied populations. Therefore, the current results should be interpreted as hypothesis-generating and intended to raise awareness, emphasizing the limitations in implementing clinical guidelines and the everyday difficulties encountered during the care of these patients. Further large multicenter studies are needed to assess the causes and consequences of the clinical overlap between HoFH and sHeFH. The additional data may support the implementation of novel therapies in selected HeFH patients with severe clinical characteristics.

## 5. Conclusions

To date, this is the first clinical study directly comparing HoFH and sHeFH patients. The overlapping serum LDL-C as well as the presence of external manifestations and cardiovascular complications in both groups emphasize the critical role of genetic testing for an accurate diagnosis. Our findings highlight the therapeutic challenges and the difficulties in achieving target lipid levels. Therefore, the availability of appropriate laboratory and molecular biological diagnostic tools, access to selective LDL apheresis therapy, and the involvement of experienced lipidologists specialized in the care of these patients are essential for developing an optimal treatment strategy. The results of genetic testing, preferences of individual patients as well as comorbid conditions should be considered during the formulation of this strategy. Furthermore, the comprehensive education of patients and their families about the nature of the disease, its expected course, potential complications and available therapeutic options are essential. Without appropriate knowledge, patient adherence may be inadequate, contributing to the failure to achieve target lipid concentrations and the development of complications, ultimately increasing cardiovascular morbidity and mortality. Providing state-of-the-art, guideline-concordant pharmacological treatment for these two special patient populations also requires broad awareness among healthcare policymakers.

## Figures and Tables

**Table 1:** Characteristics and laboratory parameters of HoFH patients (*n* = 6) at diagnosis.

	Patient 1	Patient 2	Patient 3	Patient 4	Patient 5	Patient 6
Age (years)	46	11	18	22	53	6
Gender	female	male	male	male	female	male
Total cholesterol (mmol/L)	9.50	13.20	9.80	8.20	12.09	28.11
LDL-C (mmol/L)	6.90	10.80	7.66	6.60	9.38	24.03
HDL-C (mmol/L)	2.50	0.90	1.48	1.10	1.54	2.53
Triglyceride (mmol/L)	0.50	3.20	1.47	1.60	1.53	3.40
ApoB100 (g/L)	1.72	3.17	1.88	1.69	2.23	5.5
ApoA1 (g/L)	1.99	1.17	1.49	1.51	1.58	0.66
Lp(a) (mg/L)	638	42	1204	1112	229	34
Xanthoma	-	-	-	-	-	-
Xanthelasma	-	-	-	-	-	-
Arcus corneae	-	-	-	-	-	-
CAD	ischemic heart disease	-	-	-	-	-
CAAD	-	-	-	-	-	-
Stroke/TIA	-	-	-	-	-	-
PAD	-	-	-	-	-	-
Mutations at cDNA level	LDLR c.1298C > T APOB c.662A > G	LDLR c.862G > A LDLR c.2167delG	LDLR c.2054C > T LDLR c.2140 + 5G > A	LDLR c.2054C > T LDLR c.2140 + 5G > A	LDLR c.526G > A LDLR c.2041T > C	LDLR c.1706A > G in two copies
Mutations at protein level	LDLR APOB p.Asp221Gly	LDLR p.Glu288Lys LDLR Glu723Argfs*7	LDLR p.Pro685Leu LDLR splicing variant	LDLR p.Pro685Leu LDLR splicing variant	LDLR p.Gly176Ser LDLR p.Cys681Arg	LDLR splicing variant
DLCN score	16 (8 + 8) Definite FH	17 (9 + 8) Definite FH	14 (6 + 8) Definite FH	14 (6 + 8) Definite FH	17 (9 + 8) Definite FH	17 (9 + 8) Definite FH

ApoA1, apolipoprotein A1; ApoB100, apolipoprotein B100; APOB: apolipoprotein B; CAAD: carotid artery disease; CAD: coronary artery disease; DLCN: Dutch Lipid Clinic Network; FH: familial hypercholesterolemia; HDL-C: high-density lipoprotein cholesterol; HoFH: homozygous familial hypercholesterolemia, LDL-C: low-density lipoprotein cholesterol; LDLR: low-density lipoprotein receptor; Lp(a), lipoprotein(a); PAD, peripheral artery disease; TIA: transient ischemic attack.

**Table 2:** Characteristics and laboratory parameters of sHeFH patients (*n* = 6) at diagnosis.

	Patient 7	Patient 8	Patient 9	Patient 10	Patient 11	Patient 12
Age (years)	49	59	13	45	36	49
Gender	female	female	male	female	male	male
Total cholesterol (mmol/L)	16.6	8.7	9.55	10.3	7.2	13.8
LDL-C (mmol/L)	14.1	6.3	7.85	8.08	4.87	10.3
HDL-C (mmol/L)	1.6	1.41	1.39	1.63	1.46	1.4
Triglyceride (mmol/L)	1.8	1.72	0.68	1.26	1.91	3.6
ApoB100 (g/L)	2.15	1.75	1.9	2.05	1.67	2.47
ApoA1 (g/L)	1.61	1.89	1.52	1.14	1.55	1.87
Lp(a) (mg/L)	71	1104	206	118	88	304
Xanthoma	-	-	-	-	-	tendinous xanthoma
Xanthelasma	on the right eyelid	-	-	-	-	-
Arcus corneae	-	-	-	-	-	-
CAD	CABG, PTCA	CABG	-	-	STEMI, LAD PCI	triple vessel disease, CABG
CAAD	-	-	-	-	-	plaques on both sizes without significant stenosis
Stroke/TIA	-	-	-	-	-	-
PAD	aorta plaques and calcification	-	-	-	-	stenosis of the right AFS
Mutations at cDNA level	LDLR c.(313 + 1_314-1)(1186 + 1_1187-1)dup)	LDLR c.694 + 2T > C	LDLR c.1048C > T	LDLR c.314-13_319del	LDLR c.1775G > A	LDLR c.2547 + 1 G > A
Mutations at protein level	exon 4–8 duplication	splicing variant	p.Arg350* early stop codon	splicing variant	p.Gly592Glu	splicing variant
DLCN score	19 (11 + 8) Definite FH	14 (6 + 8) Definite FH	13 (5 + 8) Definite FH	14 (6 + 8) Definite FH	13 (5 + 8) Definite FH	19 (6 + 8) Definite FH

ApoA1, apolipoprotein A1; ApoB100, apolipoprotein B100; AFS: superficial femoral artery; CAAD: carotid artery disease; CABG: coronary artery bypass graft; CAD: coronary artery disease; DLCN: Dutch Lipid Clinic Network; FH: familial hypercholesterolemia; HDL-C: high-density lipoprotein cholesterol; sHeFH: severe heterozygous familial hypercholesterolemia; LAD: left anterior descending artery; LDL-C: low-density lipoprotein cholesterol; LDLR: low-density lipoprotein receptor; Lp(a), lipoprotein(a); PAD, peripheral artery disease; PCI: percutaneous coronary intervention; PTCA: percutaneous transluminal coronary angioplasty; STEMI: ST elevation myocardial infarction; TIA: transient ischemic attack.

**Table 3:** Characteristics and laboratory parameters of HoFH and sHeFH patients.

	HoFH Patients	sHeFH Patients	*p*
number of patients	6	6	n.a.
age (years)	31.83 ± 19.5	41.83 ± 15.9	0.146
gender (female/male)	2/4	3/3	0.558
total cholesterol (mmol/L)	13.48 ± 7.4	11.02 ± 3.5	0.479
LDL-C (mmol/L)	10.89 ± 6.6	8.58 ± 3.26	0.461
HDL-C (mmol/L)	1.68 ± 0.7	1.48 ± 0.11	0.515
triglyceride (mmol/L)	1.95 ± 1.12	1.82 ± 0.97	0.845
ApoB100 (g/L)	2.70 ± 1.48	2.0 ± 0.29	0.282
ApoA1 (g/L)	1.40 ± 0.45	1.60 ± 0.27	0.381
Lp(a) (mg/L)	433.5 (42.0–1112.0)	162.0 (88.0–304.0)	0.689
xanthoma (%)	0	1 (16.7)	0.296
xanthelasma (%)	0	1 (16.7)	0.296
CAD (%)	1 (16.7)	4 (66.6)	0.790
CAAD (%)	0	1 (16.7)	0.296
PAD (%)	0	2 (33.3)	0.121

ApoA1, apolipoprotein A1; ApoB100, apolipoprotein B100; CAAD: carotid artery disease; CAD: coronary artery disease; HDL-C: high-density lipoprotein cholesterol; n.a.: not applicable; HoFH: homozygous familial hypercholesterolemia, sHeFH: severe heterozygous familial hypercholesterolemia; LDL-C: low-density lipoprotein cholesterol; Lp(a), lipoprotein(a).

## Data Availability

All data generated or analyzed during this study are included in this published article.

## Supplementary Materials

The following supporting information can be downloaded at: https://www.mdpi.com/article/10.3390/jcm14228058/s1, Figure S1: Clinical study flowchart.
