# Supplementary material for: Diagnostic and Therapeutic Challenges of Homozygous and Severe Heterozygous Familial Hypercholesterolemia from Clinical Aspect—A Single-Center Study
Source: J Clin Med. 2025 Nov 13;14(22):8058. doi: 10.3390/jcm14228058 (PMC12653393; doi:10.3390/jcm14228058)
Supplement: Supplementary file 1 [file jcm-14-08058-s001.zip › jcm-3952691-supplementary.pptx]

## Slide 1
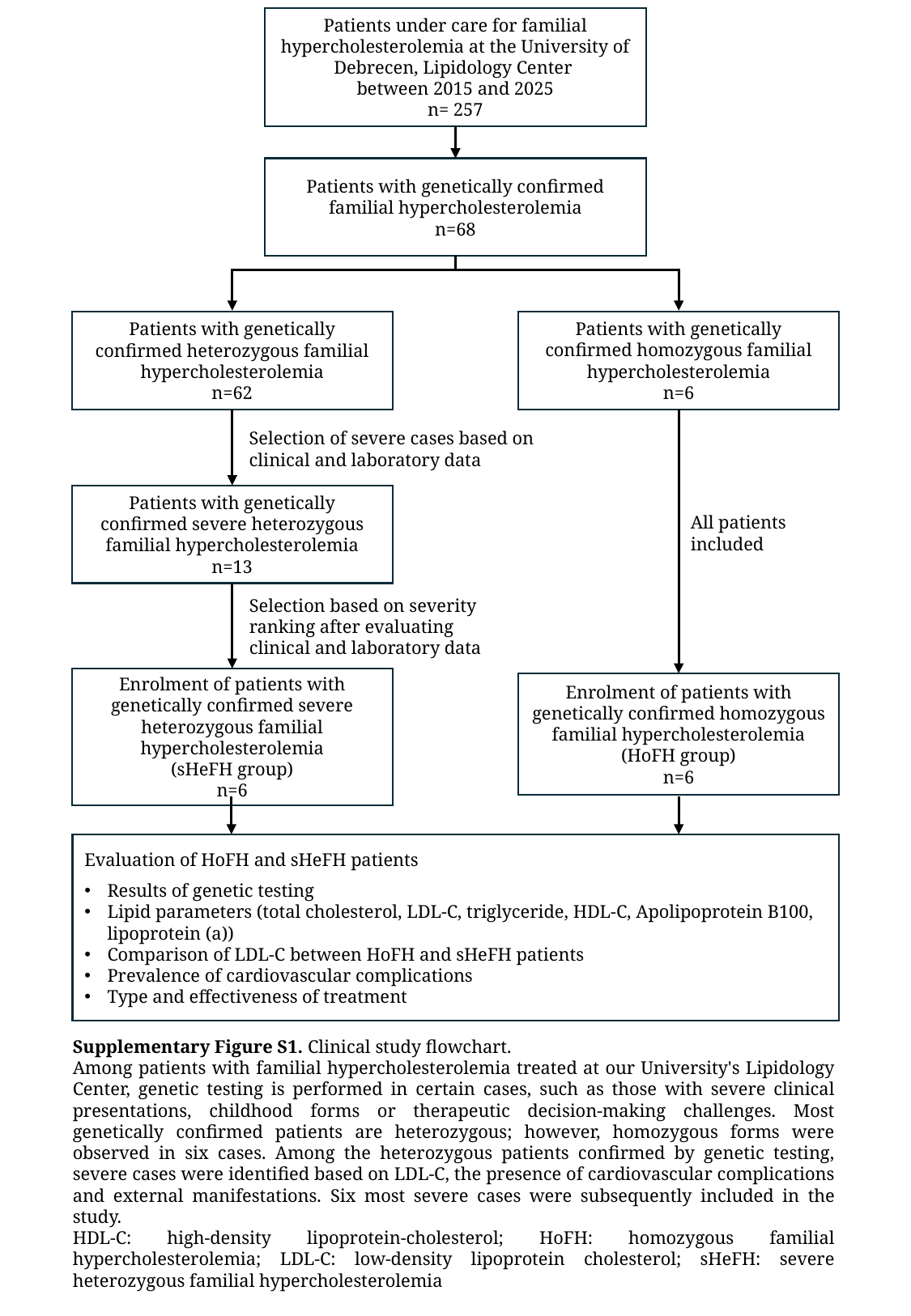

Patients under care for familial hypercholesterolemia at the University of Debrecen, Lipidology Center
between 2015 and 2025
n= 257
Patients with genetically confirmed familial hypercholesterolemia
n=68
Patients with genetically confirmed homozygous familial hypercholesterolemia
n=6
Patients with genetically confirmed heterozygous familial hypercholesterolemia
n=62
Selection of severe cases based on clinical and laboratory data
Patients with genetically confirmed severe heterozygous familial hypercholesterolemia
n=13
All patients included
Selection based on severity ranking after evaluating clinical and laboratory data
Enrolment of patients with genetically confirmed severe heterozygous familial hypercholesterolemia
(sHeFH group)
n=6
Enrolment of patients with genetically confirmed homozygous familial hypercholesterolemia
(HoFH group)
n=6
Evaluation of HoFH and sHeFH patients
Results of genetic testing
Lipid parameters (total cholesterol, LDL-C, triglyceride, HDL-C, Apolipoprotein B100, lipoprotein (a))
Comparison of LDL-C between HoFH and sHeFH patients
Prevalence of cardiovascular complications
Type and effectiveness of treatment
Supplementary Figure S1. Clinical study flowchart.
Among patients with familial hypercholesterolemia treated at our University's Lipidology Center, genetic testing is performed in certain cases, such as those with severe clinical presentations, childhood forms or therapeutic decision-making challenges. Most genetically confirmed patients are heterozygous; however, homozygous forms were observed in six cases. Among the heterozygous patients confirmed by genetic testing, severe cases were identified based on LDL-C, the presence of cardiovascular complications and external manifestations. Six most severe cases were subsequently included in the study.
HDL-C: high-density lipoprotein-cholesterol; HoFH: homozygous familial hypercholesterolemia; LDL-C: low-density lipoprotein cholesterol; sHeFH: severe heterozygous familial hypercholesterolemia
